# A qualitative study of Covid-19 effects on nutrition associated problems in recovered patients

**DOI:** 10.1186/s40795-023-00686-0

**Published:** 2023-02-13

**Authors:** Arezoo Haghighian-Roudsari, Tahereh Alsadat Khoubbin Khoshnazar, Marjan Ajami, Samira Pourmoradian

**Affiliations:** 1 Department of Community Nutrition, National Nutrition and Food Technology Research Institute, School of Nutrition Sciences and Food Technology, Shahid Beheshti University of Medical Sciences, Tehran, Iran; 2 Nursing Care Research Center, School of Nursing and Midwifery, Iran University of Medical Sciences, Tehran, Iran; 3 Food and Nutrition Policy and Planning Research Department, National Nutrition and Food Technology Research Institute, Shahid Beheshti University of Medical Sciences, Tehran, Iran; 4 Nutrition Research Center, Department of Community Nutrition, Faculty of Nutrition and Food Sciences, Tabriz University of Medical Sciences, Tabriz, Iran

**Keywords:** COVID-19, Diet, Nutrition, Qualitative research

## Abstract

**Background:**

World is currently challenging with Covid-19 pandemic. Nutritional status is a determinant factor in the treatment process and recovery for patients with Covid-19. Although a limited data is available about the effects of nutrition on this disease. Therefore, the aim of this study was to identify nutritional problems in patients recovering from Covid-19 before, during and after the disease.

**Methods:**

This qualitative descriptive study was carried out based on the specified inclusion criteria through targeted sampling of 45 patients recovered from Covid-19, 2021–2022. In-depth semi-structured individual interviews were used to collect data. Interviews were recorded, transcribed and analyzed using qualitative content analysis method and MAXQDA Software.

**Results:**

Based on the participants’ description of this stage, it can be reported that most of the individuals who were infected had no specific symptoms. Nutrition-linked problems in the main stage of the disease included troubles in tolerating foods and nutrition (e.g., fatty and solid foods), highly consumed foods in the main stage of the disease (e.g., beverages), individuals’ approaches to improve nutritional challenges (e.g., consumption of herbal teas and soft texture foods) and using supplements. The patients stated fewer nutritional problems after recovering from the disease. The most significant change included their desire to eat solid foods such as rice, bread, pasta and fast foods.

**Conclusion:**

appropriate nutrition with medication can help accelerate the recovery process of the patients, especially hospitalized patients with further severe degrees of the illness.

## Background

Currently, world is challenging with Covid-19 pandemic. The World Health Organization (WHO) has predicted that the prevalence of this disease increases, which make it the third cause of death in the world until 2030 [1]. In Iran, 7,564,252 people have suffered from Covid-19 until 31 January 2023; from whom, 144,747 lost their lives [2]. No stablished treatments have verified for this disease and in the absence of a specific treatment, solutions to prevent the disease and control its prevalence include adequate liquids to avoid dehydration and healthy diets containing nutritious foods to maintain healthy functions of the immune system [3].

The results of a systematic review have shown that the final consensus regarding the treatment protocol for Covid-19 patients has not been accurately suggested [4]. Considering the role of nutrition in the treatment protocol, it is necessary to first identify the most important nutritional problems of patients so that the results can be used in providing nutritional recommendations.

According to the researchers, nutrition management must immediately be setup for patients with Covid-19 since inadequate diets weaken the immune system [5]. Nutritional status is a determinant factor in the treatment process and recovery for patients with Covid-19 [6]. Results of several studies have indicated effects of malnutrition in the recovery process of Covid-19 patients [6, 7]. Malnutrition in the elderly with Covid-19 can make them more vulnerable, increase the severity of the disease and delay the optimal treatment [8]. Lack of nutrients and vitamins weakens the immune system functions and consequently increases the risk of other infections or exacerbates the disease if the patient is already infected [9–11]. For example, vitamin D3 includes protective roles in decreasing severity of the disease and risk of death by influenza [3, 9, 12]. Another factor affecting rate of recovery and length of hospitalization is the patients’ nutritional status and quality of diets before and during the disease. For example, diets reach in saturated fatty acids (SFAs) can lead to activation of intrinsic immune system and inhibition of the compatibility of immune system [13].

Despite all the discussions about the most effective nutritional recommendations and the role of various nutrients in preventing and helping to recover from Covid-19, it is necessary to know what nutritional problems patients are facing during the disease. However, still no studies are available on roles of diets in Covid-19 patients in Iran. Hence, this study was carried out to investigate nutritional problems in patients recovered from Covid-19 in Tehran within three stages of before the onset of the disease (latency), during the disease and after the recovery from the disease.

## Methods

This qualitative descriptive study was a part of qualitative study with a phenomenological approach on 45 patients recovered from Covid-19, 2021–2022. A qualitative descriptive approach was used specifically in cases, where data were directly collected from patients, who were experiencing the given phenomenon in a limited time within limited resources. In fact, qualitative descriptive research studies discovered phenomenon, procedure or viewpoint and the global view of those involved [14, 15].

### Selection of participants and recruitment

The study sample included patients discharged from hospitals and health centers affiliated to Shahid Beheshti Medical Sciences University and Iran Medical Sciences University, Tehran, Iran. All procedures were performed in accordance with Helsinki guideline. Purposive and convenience sampling was used for the participants on the following inclusion criteria: a confirmed history of infection with Covid-19 and recovery no matter if patients have spent their treatment period at home or in a hospital, 18–75 years of age, ability to communicate in Persian language, willingness to participate and being a Tehran resident. The names and contact information of the hospitalized patients or outpatients were collected from the hospitals and health centers. Before commence of the study, informed consent forms were collected from the participants with records of their voices. Furthermore, patients were assured about their privacy and confidentiality of their data.

### Interviews

Based on the health guidelines, interviews were carried out through phone calls for 20 min on average. The interviews were conducted using a semi-structured questionnaire with open questions where the participants explained their answers to the questions and it was possible to ask new questions during the interview based on the participants' answers. The conversation started with their verbal consent and followed by general questions on the patients’ overall statutes. Then, they were asked about their concerns on their diets before the onset of Covid-19, during the disease and after recovery from the disease. Other participants entered the study through convenience sampling. These participants were mostly those, who passed their treatment periods at homes and only used medical consultations and prescriptions. Data collection was continued until data saturation.

### Analysis

In this study, researchers reviewed the transcribed data twice to have a deeper understanding and read the contents carefully to determine and code the semantic units. Common codes were merged to extract subcategories. Then, subcategories with common aspects were compared to extract the major categories. in this study, MAXQDA Software 2020 was used to code and categorize the open codes [16].

### Data validity and trustworthiness

To assure trustworthiness of the categories, categories were reanalyzed through peer debriefing in person verbally and in written form by two other researchers, who were engaged in assessing qualitative research articles as well as other members of the academia, who were familiar with qualitative studies. After adequate discussions, suggested changes were made (if considered necessary) and then a partial consensus was reached for the final categories. In the present study, elaborate details of the methods and inductive procedures were described clearly to identify categories and support them with quotations extracted from the interviews to achieve transferability. The research team included qualitative research experts, who supervised all research processes and coding procedures. moreover, qualitative research review guidelines (RATS) was used to report the study findings structurally [17].

## Results

Data saturation was achieved after 35 interviews; however, ten further interviews were carried out to ensure that no new information were entered. The participants’ age included 39.80 y ± 13.82 with the range of 18–72 years. The participants’ characteristics are present in Table 1.

**Table 1:** Major demographic characteristics of the participants (*n* = 45)

**Variable**		***n* (%)**
**Sex**	Female	34 (75.6)
	Male	11 (24.4)
**Education**	Under high-school diploma	1 (2.2)
	Diploma	10 (22.2)
	Associate degree	11 (24.4)
	Bachelor’s degree	8 (17.8)
	Master’s degree and higher	15 (33.3)
**Occupation**	Jobless	2 (4.4)
	Housekeeper	6 (13.3)
	Employee	15 (33.3)
	University student	5 (11.1)
	Retired	3 (6.7)
	Freelancer	5 (11.1)
**Supplement use during the disease**	Zinc	4 (8.88)
	Multi-vitamins	4 (8.88)
	Vitamin C	14 (31.11)
	Vitamin D	4 (8.88)
**Place of treatment**	Home	30 (66.7)
	Hospital	15 (33.3)

Data were reported based on frequency (%)

We extracted total of 705 open codes from all the interviews. The similar codes were then categorized after deleting duplicated codes. Based on the participants’ statements, they were infected with Covid-19 because of two major reasons including: attending crowded areas such as hospitals, sport centers, wedding ceremonies, social gathering and trips and getting the virus from people around them, especially their family members, colleagues and friends. A large variety of symptoms were reported in these patients including pain in different body parts: overall body pain, headache, sore throat, stomachache, fever and chill, weakness, shortness of breath, sweating, dry nose, oral and nasal burning and backache.

Nutritional problems of outpatients and inpatients were almost similar, with the difference that the severity and duration of the problems, especially anorexia and food intolerance, were higher in hospitalized patients. The problem of anorexia in people who had anosmia lasted longer because it also affected their taste.

Based on the research objectives, the concepts obtained from the open and axial coding are put into three categories: latent, main and recovery stage of the disease. Table 2 presents the main themes and sub-themes and some quotes of the participants.

### Latent stage of the disease

**Table 2:** Summary of the major themes, sub-themes and quotes from the participants

***Main Themes***		**Sub-themes**	***Some Quotations***
***latent stage of the disease***			*“As we thought it is a cold, and in cold our nose gets stuffed, and the sense of smelling decreases a bit, we were not that much concerned about it. Though during the disease, I had no sense of taste or smelling, and it was so bad! I mean I couldn’t tell what I was eating at all! After a month, little by little, I could have my sense of smell and taste back” (Female, 26 years old)* *“2–3 days before referring to the doctor and confirming my infection with coronavirus, my sense of smelling was decreased. I could only feel very strong smells. I was also nauseated which got worsened after my infliction, too. Maybe for example a week before getting corona virus, my appetite had decreased a bit” (Female, 22 years old)* *“A couple of days before, my appetite had decreased. I had some digestion problem, too” (Male, 30 years old)* *“Before my coronavirus period, I only had pain in my hands and feet” (Male, 40 years old)*
***Main Stage of the disease***	***Trouble in tolerating foods and nutrition***	**Fatty foods, Solid foods with hard tissue**	*“I had less tendency to eat some foods like fast foods, any sauces or fried foods. I actually could not stand fast foods or those foods that had sauces or mozzarella cheese” (Female, 49 years old)* *“Based on my physical conditions, I couldn’t eat fatty or heavy foods. I Couldn’t eat fried foods and oily foods, either! If I ate, I felt so full and heavy and had pain in my stomach!” (Female, 53 years)* *“I felt nauseated when I ate rice. I felt sick. Even when I ate less than one serving of rice, still I vomited and had severe nausea and stomachache! “Up to two weeks I could only eat soups. It was hard for me to tolerate solid foods” (Female, 22 years old).”* *“I could not eat bread, as it was harder. It was solid. I couldn’t eat Abgousht (a heavy meat and beans pottage), either as it was so heavy and fatty!” (Male, 50 years old)*
		**Preservative-containing foods**	*“I could not use any allergy or cough stimulant foods like flavors!” (Female, 47 years old)* *“I could not eat any food that had tomato paste on it!” (Female, 22 years old)*
		**Dairies and watery foods**	*“For breakfast, if I ate cheese and walnut, my coughing would get worse” (Female, 28 years old)* *“Having cold water, Doogh (Yoghurt drink) and yoghurt generally gave me a chill. For this, I used to avoid them” (Female, 44 years old)* *“Watery foods were harder to eat, I think I had difficulty digesting them” (Female, 22 years old)*
	***Highly consumed foods in the main stage of the disease***	**Beverages**	*“I felt like drinking more liquids. My liquid consumption was higher especially water and natural fruit juices like apple juice and carrot juice” (Female, 49 years old)* *“I used to drink a lot of hot beverages like tea and herbal drinks. I drank a lot of black tea with ginger and brewed herbs” (Female, 44 years old)*
		**Mixed dishes**	*“Watery foods were good. From the second week, I got out of bed and made myself rich mixed soups like those that can be eaten through Gavage (Food given to the patient through the nasogastric tube)” (Female, 40 years old)*
		**variety of Fruits**	*“Though my appetite was very low, I had developed a strong craving for fruits like nectarine, apple and black plum” (Female, 49 years old)* *“I loved eating sweet lemon” (Female, 53 years old)*
	***Patients’ approaches and solutions to improve diets in the main stage of the disease***	**Consumption of variety of beverages and herbal teas**	*“We used to drink fruit juices, orange juice, carrot juice and apples. I used to brew herbal drinks with chamomile and thyme” (Female, 34 years old)* *“We mostly used to drink brewed thyme, cinnamon and ginger. We drank a lot of liquids like carrot juice, orange juice and herbal drinks” (Male, 29 years old)* *“More herbal drinks, more honey. I used to drink brewed thyme, Damask rose and cinnamon. Before Corona, I used to drink them maybe once a month. Though during COVID, I used to drink it twice a day. These were my remedies when I caught cold” (Male, 72 years old)* *“In general, warm and watery foods, and brewed thyme, apple mint, ginger and cinnamon are so good. And fruit juices, especially carrot juice with apple juice” (Female, 39 years old)*
		**Eating soft texture foods**	*“Soup or Aash or starch porridge every day or every other day! In general, I mostly used liquids and soft foods” (Male, 50 years old)* *“I mostly used small but rich meals. I tried to include protein and vegetables in my diet” (Female, 45 years old)*
		**Increased fruit and vegetables consumption**	*“We definitely tried to eat citrus family fruits as they have vitamins and made me feel better, and my throat was not sore for a while after eating them. In fact, we mostly ate lemon, grapefruit, and some oranges” (Female, 44 years old)* *“I tried to add lemon juice or green grape juice to the foods to make them more appealing with the sour taste as we mostly had soups or pottages. I tried to put various vegetables in the soups to at least use their vitamins” (Female, 44 years old)*
		**Consuming foods that are conventionally believed to be beneficial**	*“I made myself eat anything as if I had to! I even tried to make my diet better and richer by, for instance, eating quail and quail egg, more walnuts, more herbal drinks, more honey, *etc*. I used to drink brewed thyme, damask rose and cinnamon” (Female, 44 years old)* *“My relatives brought us foods like quail, cooked veggies, carrot juice and the herbal teas that they thought they are beneficial for me” (Female, 47 years old)* *“Relatives made me quail extract that I used to eat, and lamb leg stew, organic fruit juices like carrot, orange and sweet lemon, …” (Female, 45 years old)* *“Almost every day, I made myself eat a mix of ground apples, saffron, damask rose extract and honey as my daily fruit unit. I ate lamb leg stew on the first three days of my infection with the disease” (Female, 28 years old)* *“Fruit juice, apple juice, carrot juice, quail and quail egg! There were even changes in the way we used them. For instance, four to five times a week, we had Fesenjoon (a Persian stew made with lots of walnuts, pomegranate juice, and meat or chicken). It turned to be my major meal because of the walnuts. I used to eat dates and walnuts with herbal drinks, too. I asked people to get some black seeds for me. I ground them and ate them with honey. I could find the Royal Gel, the food of the queen bee, with the help of an acquaintance who lives near Sarab (Name of a city in Ardabil province.)” (Male, 72 years old)*
	***The supplements used***		*“Taking vitamin pills, we tried to improve our appetite and bring back our appetite or the sense of our willingness to eat!” (Female, 18 years old)* *“Multivitamins, too. I used to take them every day during the disease. Before that I used to take them every other day” (Male, 72 years old)* *“Vitamin C, the effervescent tablet is awesome for Corona patients. My doctor had prescribed me Zinc tablets and Vitamin D, too” (Male, 38 years old)*
**Nutritional issues in recovery stage**			*“I mostly like foods like rice, egg, solid foods. I generally feel bad eating watery foods except Aash. Even the idea that I had eaten them makes me feel sick” (Female, 44 years old)* *“The first food after recovery was fast food because we were forbidden to eat spicy and fast foods during the disease. As soon as the period was over, I wanted to go get a lot of pizza, fried chicken, and the like” (Female, 26 years old)* *“I felt like eating Abgousht as it was both warm and rich and was one of my favorites, too. Sometimes I liked to fry potatoes, though as I was worried it causes coughing, I made them in Microwave oven with a little bit of oil” (Female 44 years old)*

Based on what the participants described, this stage was like the fire smoldering under the ashes as changes occurred in the body, while no significant clinical expressions were manifested. According to the participants’ description of this stage, it can be understood that most of the patients had no specific symptoms such as vomiting, loss of appetite and changes in the taste and smelling senses with no changes their diets. Furthermore**,** a number of the participants stated symptoms before the major symptoms of the disease. These symptoms included loss of appetite, decreased sense of smelling, vomiting and pain in hands and legs.

### Main stage of the disease

Coronavirus patients had their biggest nutritional, physical and mental challenges in this stage. Based on the participants’ statements, their nutritional problems could be divided into the following subcategories:

### Troubles in tolerating foods

According to the participants, their most significant nutritional problem in this period included troubles in tolerating some foods due to lack of appetite, nausea and sometimes stomachache. These foods could generally be categorized as follows:

The most important challenge for most participants included use of fatty foods, solid foods such as fried foods, dry foods, heavy foods, fast foods, rice, pasta, bread, meat, chicken, sausages, salami and foods such as Olivier salad. Some participants complained of foods with preservatives, including all sorts of flavors and tomato pastes and some other participants reported problems with the consumption of dairy products, especially milk.

### Highly consumed foods in the main stage of the disease

Food items were used more frequently by these patients considering their symptoms and difficulties in taking especial foods. A variety of drinks, fruits and mixed foods were consumed mostly, comparing to the past.

As mentioned in the section about highly consumed foods, the consumption pattern that can be deduced from this group of foods is identifying the ones that were used based on the patient’s choices and preferences. In the period of entangling with the disease, patients preferred a variety of boiled, soft-texture low-fat foods such as soups and pottages, barbecued or boiled chicken and meat, drinks and beverages such as water, fruit juices and herbal drinks and fruits, especially citrus fruits.

It can be concluded that beverages included the highest consumption rate as most participants reported use of various beverages in their diets. As the doctors and medical providers highly recommended continuous hydration, effects of these recommendations were clearly significant in the patients’ diets. Of drinks, tea and various herbal drinks were highly popular. In addition to drinking fruit juices, patients significantly increased eating fruits. Various types of citrus and seasonal fruits were included in their daily diets. Another group of highly consumed foods included mixed dishes that contained a larger spectrum of food types. According to the participants, various types of soups and ash (a traditional veggie, grain and bean pottage) and protein products included the highest consumption rates in these patients for various reasons such as physicians’ recommendations.

Some other participants stated that they had increased their protein products consumption probably for some reasons as they might have thought these foods are nutritious and would affect recovering from the disease.

### Patients’ approaches and solutions to improve diets in the main stage of the disease

Based on the participants’ statements, patients applied food and non-food linked solutions for their problems or their family members based on their personal experiences or experience from other people. These solutions included drinking various liquids and eating soft-texture foods, fruits, vegetables and conventionally favored foods by the general public, which were sometimes effective in their treatments. Since these solutions were not sometimes preferred by the patients, they used the solutions only because solutions were suggested to help their recovery. The most important examples of this group; from which, many participants had at least one included brewed herbal teas such as mint, thyme and ginger teas, used mostly as cures of coughing. A variety of fresh fruit juices were used, especially carrot and orange juices, as well as water even if the patients did not willing to and a mixture of lime juice and honey were highly prioritized. As soft-texture foods were easier to eat compared with solid foods, especially in the case of severe coughing in patients, use of soft-texture foods was preferred. Participants expressed how better they felt, when they ate warm foods with no solid textures.

One of the patients’ approaches included increased consumption of fruits and vegetables, comparing to their routine diets. As a strategy to improve their diets, participants added condiments such as lemon and green-grape juices to their diets due to the significant improvements in the food general tastes as well as their vitamin contents.

Foods conventionally believed as beneficial were used based on other people recommendations or personal experiences. Some participants reported use of further rich foods to strengthen their bodies; however, patients had various interpretations of the rich foods. Moreover, participants reported use of quail for this disease. It is commonly believed that eating quail can help recovering from Covid-19, verified by high consumption of quails. Use of foods such as mutton muscles and lamb shinbones in soups to make the food richer, eat of walnut and consumption of various fruit juices such as carrot, orange and apple juices and a mixture of honey with other foods such as herbal drinks, fruits, lemonades and teas were more frequently reported by the interviewees.

### The supplements used

In the present study, patients used supplements frequently more than that they did conventionally. Participants reported the highest supplement consumption in their infection period for vitamin C, zinc, vitamin D and multivitamins.

### Nutritional issues in recovery stage

People experienced a fewer nutritional problems and challenges after recovering from the disease, when their symptoms decreased. Based on what the participants’ asserted, they experienced changes in their food preferences such as solid foods. Foods such as rice, bread, pasta, abgoosht (a traditional meat pottage) and fast foods appeared on their diet lists. Since the participants did not have much varieties in their food consumption during the disease period, they had a further desire to eat foods that were previously avoided.

## Discussion

Previous reports indicated that infectious disease could be an important cause of weight loss, and impaired physical growth. Also, it was a common determinant factor of severe malnutrition and death [18]. In the case of COVID-19 the effect of disease on dietary habits, was unknown and most of the published studies on nutrition and Covid-19 were limited to the effects of lockdown and stay-at-home on eating habits [19, 20].

In the present study, gastrointestinal symptoms such as nausea and losses of appetite and smell were reported as the most important nutritional problems or factors affecting people's eating. Though in retrospective case–control research that compared dietary habits in patients with COVID-19 and healthy participants no significant differences in dietary habits between patients with Covid-19 and healthy participants were found in the previous year [21] that the present results verified these findings. These findings could be justified with the fact that the infection must enter the body to activate several intercellular reactions to change the satiety-hunger cycle and alter dietary habits and food preferences. An increasing number of studies have reported digestive problem manifestations such as loss of appetite, diarrhea, vomiting and abdominal pain in patients with Covid-19 [20, 22, 23]. Findings of this study showed that the most common nutrition problems in patients with Covid-19 during the disease period included nausea, vomiting, food intolerance and loss of appetite, which made them unable to eat well, compared to the period before the disease.

In a recent meta-analysis by Mao et al., the pooled prevalence of digestive symptoms in patients with Covid-19 was 15%. Prevalence of nausea, loss of appetite and abdominal pain were 6, 21 and 3%, respectively [24]. On the first few days after the infection, SARS‐CoV‐2 could induce acute nausea and vomiting by triggering the release of hormones from enteroendocrine cells (EECs) in the mucosa of the upper gastrointestinal tract (GIT) [22]. In an online survey by Chaaban et al. on patients with Covid-19, 86% of the patients reported loss of appetite, compared to that they did before Covid-19 [23]. Loss of appetite with losses of smell and taste included greater effects on food intakes in the patients. Based on the health belief model, beliefs are crucial determinants for choosing effective health promotion behaviors and dietary intakes during several diseases. Moreover, it is helpful in designing and implementing health communication initiatives [25, 26]. Hence, questions were asked in the present study to investigate home remedies and special food and eating-linked restrictions during Covid-19. In the present study, patients reported intolerance for solid foods and increased eating of fruits, vegetables, nuts, natural sweets such as date and honey, herbs and hot liquid foods such as soups and drinks such as water.

According to the statements of the participants, their food choices were more subject to the conditions of the disease and what they heard from the medical community, virtual space and the experiences of people who had already contracted the disease. More consumption of natural fruit juices, ginger and herbal teas that were popular in traditional Iranian medicine, were consumed a lot during the covid epidemic.

As far as the authors know, no studies have investigated food intakes in patients with Covid-19. Study of dietary intakes in lockdown period showed that the consumption of fruits and vegetables significantly increased in adults from Republic of Ireland, United Kingdom, United States of America and New Zealand [27]. A study in Kuwait showed no significant changes in consumption of red meats, chickens, fats, milks, breads, fruits and vegetables before and during the pandemic, except for fish and seafood [28]. Another study during the Covid-19 lockdown in Italy indicated that people’s diet quality improved with the Mediterranean diets [29]. Additionally, they preferred to eat boiled and steamed foods. These preferences might be due to their beliefs regarding the ameliorating effects of boiled and steamed foods on cough, sore throat and digestive problems [30]. However, participants had trouble ingesting foods with additives and those containing tomato pastes. These effects might be explained by the allergic reactions, which activated the release of mediators in the airways or by the presence of histamine or other mediators in food additives and spices that could constrict the airway smooth muscles directly or via reflexes [31].

During the first wave of Covid-19 outbreak, use of several herbals and traditional medicines and supplements widely increased as a possible preventive method to decrease possibility of infection with SARS-CoV-2 or as a method to decrease complications and several symptoms of the disease such as cough and sore throat [32]. Most scientific evidences have rejected the idea that such specific foods and supplements can inhibit viral infections [32].

In a previous study, participants claimed that they used several types of herbal drinks in addition to routine medications based on their own experiences and recommendations from their relatives and friends, who were previously infected. In a recent study by the current authors, boron supplementation could improve clinical and biochemical parameters in patients with Covid-19 [33]. Another nutritional attitude, which most patients reported during the Covid-19, included the hot–cold nature of foods. For instance, most of the patients avoided dairies and poultry meats but increasingly used honey, royal jelly and quail meat and eggs. In the culture of most countries in south Asia and Middle East such as China, Korea and Iran, foods are categorized with hot or cold natures, regarding natural tempers associated with the foods and people eating them. In their beliefs, hot–cold balance in the body is crucial for maintaining the health [34]. A few studies have suggested that hot–cold balance may play roles in regulating inflammatory markers [26, 35, 36]. In Iranian tradition, foods and herbs serve as medicine for curing diseases. These herbs such as ginger, sweat lemon, citron, myrtle and lavender include antipyretic, antitussive, anti-inflammatory, antioxidant and antimicrobial characteristics [37, 38].

The present results showed that most of the participants used vitamin and mineral supplements even six months before their infection with Covid-19, revealing their beliefs in the protective and immune-enhancing effects of these supplements. However, vitamin D, zinc and vitamin C were prescribed for most of the patients as a part of their treatment plans during the disease. A similar study in Saudi Arabia reported that dietary or herbal supplements intake increased patients’ immune response to Covid-19. Moreover, consumption of these supplements decreased risks of hospitalization [25].

In the recovery period, ameliorated disease symptoms such as nausea, vomiting and increased appetite changed the patients’ dietary intakes. As presented in results section, patients reported their willingness to solid foods such as rice and unwillingness to watery foods and soups. Their beliefs in feeling weak and low-energy after the illness also encouraged them to eat further nutritious and high-calorie foods such as pottages during the recovery.

Nutrition support is a vital part of the treatment protocols and early recovery from the infectious diseases such Covid-19. Lack of nutrition support and malnutrition increase duration of hospitalization and recovery time [32, 39–42]. Optimized nutrition statuses enhance the immune system through the adaptation of cellular signaling and gene expression. Strengthening the immune system characterizes one supportable way to improve the likelihood of surviving from Covid-19 [43]. The results of a nation-wide study by Cobre et al. revealed that consumption of eggs, fish, seafood, fruits, meats, milks, starchy roots, vegetables, nuts and vegetable oils played positive effects on the recovery from Covid-19. They concluded that diets rich in proteins and healthy fats (olive and fish oils) were beneficial, while drinking alcohol included negative effects on the disease [40].

The most important strength of the study was that the nutritional problems of the patients with covid were extracted from the statements of the same people who had the disease and recovered. This study was conducted during the peak of the covid epidemic in Iran, it was not possible to have a face-to-face interview due to the fear of the spread of the disease, and this issue was considered as a weakness of the qualitative interview.

## Conclusion

In general, it seemed that the most important nutritional challenges in patients with Covid-19 occurred in the period of their infection. However, problems in the latent period and after the recovery were not serious or difficult. Based on the results of this study, types of foods and continued consumption of fluids helped the patient recover and strengthen their immune system. In conclusion, appropriate nutrition with medical prescriptions can help accelerate the recovery process of the patients, especially hospitalized patients with severe degrees of the illness.

## Data Availability

The datasets used and/or analyzed during the current study available from the corresponding author on reasonable request.

## Abbreviations

WHO: World Health Organization
SFAs: Saturated fatty acids
RATS: Relevancy, appropriateness, transparency, and soundness
